# Impact of air exposure and annealing on the chemical and electronic properties of the surface of SnO_2_ nanolayers deposited by rheotaxial growth and vacuum oxidation

**DOI:** 10.3762/bjnano.8.55

**Published:** 2017-02-27

**Authors:** Monika Kwoka, Maciej Krzywiecki

**Affiliations:** 1 Institute of Electronics, Silesian University of Technology, Akademicka 16, 44-100 Gliwice, Poland; 2 Institute of Physics – CSE, Silesian University of Technology, Konarskiego 22B, 44-100 Gliwice, Poland

**Keywords:** Fermi level position, RGVO nanolayers, rheotaxial growth and vacuum oxidation (RGVO), surface chemistry, tin-oxide electronics, X-ray photoelectron spectroscopy (XPS)

## Abstract

In this paper the SnO_2_ nanolayers were deposited by rheotaxial growth and vacuum oxidation (RGVO) and analyzed for the susceptibility to ambient-air exposure and the subsequent recovery under vacuum conditions. Particularly the surface chemistry of the layers, stoichiometry and level of carbon contamination, was scrutinized by X-ray photoelectron spectroscopy (XPS). The layers were tested i) pristine, ii) after air exposure and iii) after UHV annealing to validate perspective recovery procedures of the sensing layers. XPS results showed that the pristine RGVO SnO_2_ nanolayers are of high purity with a ratio [O]/[Sn] = 1.62 and almost no carbon contamination. After air exposure the relative [O]/[Sn] concentration increased to 1.80 while maintaining a relatively low level of carbon contaminants. Subsequent UHV annealing led to a relative [O]/[Sn] concentration comparable to the pristine samples. The oxidation resulted in a variation of the distance between the valence band edge and the Fermi level energy. This was attributed to oxygen diffusion through the porous SnO_2_ surface as measured by atomic force microscopy.

## Introduction

For many years, tin dioxide (SnO_2_) has been widely used as the active material for resistive-type gas sensors for oxidizing and reducing gases [1], thin transparent electrodes and barrier layers in solar cells [2]. This is related to its high and variable electrical conductivity in the range of 10^0^ Ω^−1^·cm^−1^ to 10^2^ Ω^−1^·cm^−1^ due to the existence of free electrons in oxygen vacancies. This effect has been widely applied for the construction of prototypical gas sensors devices with both thick and thin films [3–8].

The abovementioned properties of the SnO_2_ thin films strongly depend on their deviation from stoichiometry, the amount of dopants/impurities and the microstructure of the films. All these properties can be affected by the deposition method, and by post-deposition processing.

Lately, the mainstream of worldwide research is focused on further improvement of the performance of sensors based on nanostructured metal oxides (including one-dimensional) [1,9–11]. Nevertheless, one of the most popular technologies for the fabrication of SnO_2_-based sensors is the thin-film technology. This is because of basic advantages such as simplicity, repeatability, and low power consumption [5–8]. Despite many years of research, there are still a number of unsolved limitations to SnO_2_ resistive-type thin-film gas sensors, such as small sensitivity caused by low internal surface or very long response and recovery times under typical working conditions. Moreover, a crucial issue is the control of the prolonged exposure effects and contamination with carbon compounds and water vapor, because carbonaceous/water species are saturating (hence eliminating) the active surface sites available for any adsorbents to be detected and may cause an alteration in local subsurface electronic structure of the material.

This is why there is natural tendency to search completely new or to modify recently developed technological methods. The preparation of novel SnO_2_ thin films with properties tuned to a particular application (e.g., by control of stoichiometry) can be a way to eliminate the disadvantages and limitations mentioned above.

In our recent studies [12] we have proposed a modification of the rheotaxial growth and thermal oxidation technology, which is one of the most commonly used approach for the preparation of very sensitive SnO_2_ thin films [13–16] that yield the highest sensor response to nitrogen dioxide [17]. Our current approach is focused on the rheotaxial growth of Sn single nanolayers under ultrahigh-vacuum conditions combined with the simultaneous in situ vacuum oxidation (RGVO), which results in SnO_2_ nanolayers of controlled nonstoichiometry/stoichiometry depending on the intended application.

This paper presents the X-ray photoelectron spectroscopy (XPS) results on the variation of surface chemistry and electronic properties of RGVO SnO_2_ nanolayers after exposure to air and subsequent UHV annealing (outgassing) for verification of their behavior under real working conditions of a semiconductor-based device. Further, the reversibility of the exposure effects is carefully analyzed. The impact of the exposure is being scrutinized on the basis of the changes in the chemical/electronic structure of the layers as well as the post-exposition-related contamination. The studies are augmented with surface topography investigations using atomic force microscopy (AFM) in order to check the porosity of the resulting layer.

## Experimental

The RGVO SnO_2_ nanolayers (20 nm, quartz microbalance controlled) were deposited under UHV conditions (system base pressure: 3·10^−9^ mbar) by thermal evaporation of Sn pellets (KJLC^®^) from a resistively heated source on Si(100) substrates (Bosch GmbH, n-type, P-doped, 5–10 Ω·cm) maintained at a temperature of 265 °C at an oxygen partial pressure of 10^−4^ mbar for 2 h. In order to improve their stoichiometry, an additional in situ vacuum oxidation was performed at a chosen optimal partial pressure of 10^−2^ mbar for the next 2 h (10^8^ L). The samples were then examined by using XPS. At the next step the samples were exposed to dry air with a relative humidity of 50% and a constant temperature of *T* = 21 °C for 72 h, which ensured the saturation of the exposure effect. Then the samples were re-examined by XPS. At the final step the air-exposed samples were annealed (outgassing) at 265 °C (the standard substrate temperature for deposition of the RGVO SnO_2_ nanolayers) under base UHV conditions for 2 h. After this procedure the samples were again examined by XPS.

XPS measurements were performed using a SPECS spectrometer (base pressure about 10^−9^ mbar) equipped with an X-ray XR-50 source (Al Kα) and a hemispherical analyzer (PHOIBOS-100). The pass energy was set to 80 eV for the survey spectra and 10 eV for recording the individual core-level spectra. The binding energy (BE) scale of recorded spectra was calibrated to the Au 4f_7/2_ (84.0 eV [18]) peak. XPS data were analyzed by curve fitting using the CASA^®^ XPS software. The estimated uncertainty in determining the position of a particular component in XPS measurements was within 0.07 eV. Quantitative analysis, including the determination of component ratios, was done with the use of atomic sensitivity factors (ASF) and procedures described in detail in [12,19–21].

AFM studies have been performed using the PSIA XE-70 scanning microscope working in contact mode. Budget Sensors monolithic silicon probes ContAl-G (resonance frequency 13 kHz, force constant 0.2 N·m^−1^) were used. The XEI^®^, PSIA and Gwyddion^®^ image processing software allowed us to correct sample inclination and distortions caused by the *z*-scanning stage. No other corrections to the images were made. For quantitative topography analysis the Gwyddion^®^ software was also used. As the measurement of surface roughness, the root mean square (RMS) of roughness was quantified, where the root mean deviation from a plane was analyzed. Surface area estimation was performed by triangulating the surface (as stated in the algorithm description) and summing up their area to obtain the total area. Further details on the methodology can be found elsewhere [22–23].

## Results and Discussion

### Surface chemistry

**Figure 1:**
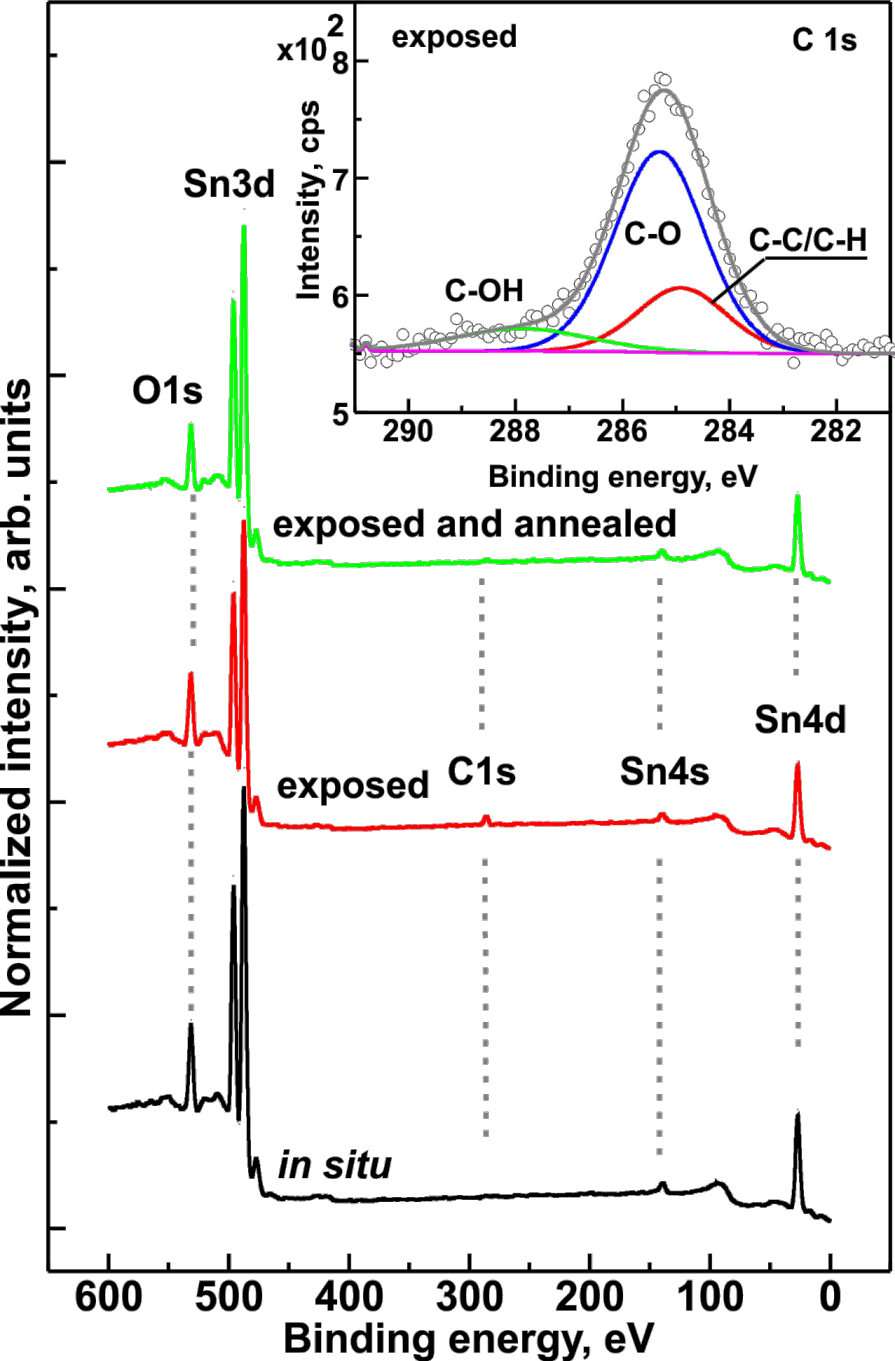
XPS survey spectra with the main core-level lines of RGVO SnO_2_ nanolayers (pristine (“in situ”), after exposure to air (exposed) and after subsequent UHV annealing (exposed and annealed)). Inset: decomposed C 1s region for the air-exposed sample showing the main constituents of the carbon contamination.

Figure 1 presents the XPS survey spectra of RGVO SnO_2_ nanolayers (pristine, after exposure to air and after subsequent UHV annealing).

For the pristine layers only O 1s, double Sn 3d, Sn 4s and Sn 4d core lines were detected. After exposure to air apart from an intensity decrease of the main core lines, an evident C 1s peak appeared. This means that after air exposure the RGVO SnO_2_ nanolayers were covered with C contaminations originating from ambient air. The inset in Figure 1 presents the decomposition of the C 1s XPS region conducted for the recognition of carbon contaminations. The C contamination comes from adventitious carbon (C–C and C–H components), and from a C–O component, which most likely originates from adsorbed CO/CO_2_, and from C–OH groups [24–26]. However, the overall detected C 1s signal is at a level of about 1.3 (peak-to-noise), which means that the carbon contamination of the RGVO SnO_2_ nanolayers is lower than moderate. This is of significant importance for the potential application as gas sensor material [16,19], because it shows that even after air exposure the layers kept their high purity and the available adsorption sites are not occupied mainly by carbonaceous species.

After subsequent UHV annealing of the air-exposed samples the XPS survey spectrum is very similar to that observed for the pristine RGVO SnO_2_ nanolayers. Most importantly, after annealing the C 1s peak almost disappeared. It could indicate that the C contamination is mainly the physisorption of carbonaceous species from ambient air leaving only small possible contribution for chemical adsorption processes. The latter can be precisely detected by careful investigation of the O 1s and Sn 3d energy regions.

**Figure 2:**
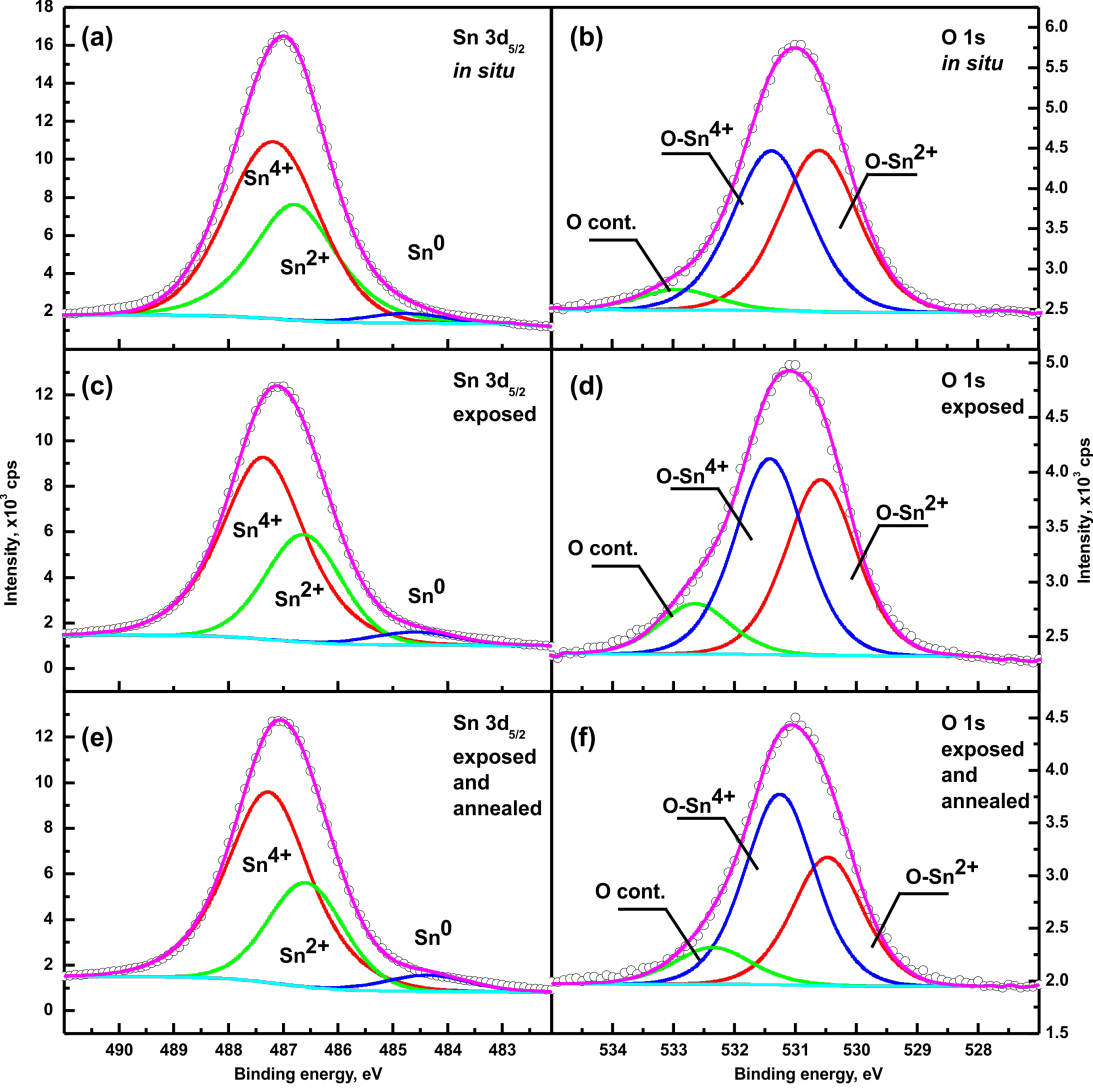
The decomposed Sn 3d_5/2_ and O 1s XPS lines of RGVO SnO_2_ nanolayers for pristine (“in situ”) samples (a and b), after air exposure (c and d), and after their subsequent UHV annealing (e and f). The cyan line in all panels refers to background level fitted with a Shirley function.

In order to conduct a more precise analysis of the stoichiometry/nonstoichiometry of the RGVO SnO_2_ nanolayers after technological procedures (the main aim of our research) the Sn 3d_5/2_ and O 1s core lines were decomposed as shown in Figure 2. Each peak is represented by a sum of Gaussian (70%) and Lorentzian (30%) lines, while the secondary electron background was subtracted utilizing the Shirley function (cyan line in Figure 2). The results of the quantitative analysis carried out using ASF [27] are summarized in Table 1. The overall stoichiometry, i.e., the relative [O]/[Sn] concentration varies from 1.62(6) for pristine samples to 1.80(6) for RGVO SnO_2_ nanolayers exposed to air and then decreases to 1.65(6) for the annealed samples. Consequently, we state that the results clearly point to a successful recovery of the material to its initial state.

**Table 1:** Relative concentration of main elements of RGVO SnO_2_ nanolayers estimated on the basis of a recently used ASF procedure [27] as well as on the basis of the decomposed of the O1s and Sn3d_5/2_ spectral lines shown in Figure 2.

sample	relative [O]/[Sn] concentration based on relative intensities of Sn 3d_5/2_ and O 1s XPS lines and ASF procedure	relative [Sn^4+^/Sn^2+^] concentration calculated on the base of area of decomposed XPS lines	
		(O–Sn^4+^/O–Sn^2+^)	(Sn^4+^/Sn^2+^ + Sn^0^)

pristine	1.62(6)	1.05	1.64
exposed to air	1.80(6)	1.17	1.71
exposed to air and annealed	1.65(6)	1.46	1.71

For all of the RGVO SnO_2_ nanolayers strong components related to Sn^4+^ and XPS Sn^2+^ at energies of 487.2 eV and 486.6 eV, respectively, were observed in the Sn3d_5/2_ spectral lines. However, there was also a small additional component visible at a binding energy of 485 eV, which could be attributed to elemental tin Sn^0^. This finding indicates that these nanolayers were not completely oxidized.

In the XPS O 1s spectral lines, apart from the main expected components related to the O-Sn^4+^ as well as the O-Sn^2+^ components at 531.2 eV and 530.4 eV, respectively, a small additional component was visible at a binding energy about 533 eV. We suspect that it could be attributed to O=C or C–OH contaminants, likely existing at the surface of RGVO SnO_2_ nanolayers after exposure to air [28] as well as to partially ionized –OH groups [26] originating from, e.g., dissociated water. Such water dissociation was predicted by Xu et al. [29], where the splitting of water on metal oxide surfaces was modelled and investigated.

The small relative decrease of overall signal intensity after air exposure could be related to C contamination at a level of [C]/[Sn] ≈ 1.0. This analysis confirms that the carbon contamination was kept at a low level after exposure. Based on the decomposition of the XPS Sn3d_5/2_ line (Figure 2a), one can easily derive for pristine RGVO SnO_2_ nanolayers that the relative (Sn^4+^/Sn^2+^ + Sn^0^) concentration increased. It shows that the in situ RGVO nanolayers consists of a mixture of SnO and SnO_2_ with only weak domination of the latter one. This is in good agreement with the decomposition of the XPS O1s line (Figure 2b) in which the (O-Sn^4+^/O-Sn^2+^) ratio is 1.05.

After exposure to air, the overall relative [O]/[Sn] concentration (see column 2 of Table 1) increased by about 10%. This finding agrees well with the information obtained after the decomposition of the respective O 1s and Sn 3d_5/2_ spectral lines shown in Figure 2c,d. It confirms that after air exposure the nanolayers still consisted of a mixture of SnO and SnO_2_, but with a strong domination of the latter. According to Figure 2a and Figure 2c the amount of elemental tin did not change significantly. Therefore, one can conclude that the variation of the different tin oxidation states takes place mainly between Sn^2+^ and Sn^4+^. This conclusion is also confirmed by analysis of O 1s region where O-Sn^4+^ signal was increasing after exposure. Of course, a contamination-related signal was slightly emerging as well.

After subsequent UHV annealing of the air-exposed RGVO SnO_2_ nanolayers the overall relative [O]/[Sn] concentration reached a value comparable to the pristine samples. However, looking at the respective O 1s and Sn 3d_5/2_ spectral lines shown in Figure 2e,f it is easy to observe that i) the SnO_2_ phase became more dominant and ii) the amount of contaminants decreased. The first finding led us to conclusion that the quantity of oxygen adsorbed during air exposure was diffusing towards deeper regions of the layer and oxidized additional tin during the annealing process. This is visible especially in the (Sn^4+^/Sn^2+^ + Sn^0^) ratio (see Table 1). In contrast, the general [O]/[Sn] concentration was reduced almost to the level of the pristine samples. The latter shows, that the part of the oxygen that did not diffuse into the layer was either physisorbed or weakly chemisorbed at the layer surface (reversibly chemisorbed). The change in the O 1s spectrum after exposure to air most likely hints at the second alternative.

The (O-Sn^4+^/O-Sn^2+^) ratio exhibited a stable tendency; i.e., each consecutive treatment was increasing the presence of higher oxidation states of the elements within the spectra. This confirmed the fact of additional vacuum oxidation of the RGVO SnO_2_ and indicated that the change of oxidation state was irreversible after UHV annealing. The last issue to be discussed within Figure 2 is the behavior of contaminations related to carbon and water vapor. The increase of the contaminations in the O 1s energy region introduced by air-exposure process was not completely reversible. This fact also led us to conclusion that carbonaceous and water-related species are partially strongly chemisorbed and partially physisorbed or weakly reversibly chemisorbed. Especially the water-related adsorbates are most probably a consequence of a partial dissociation of water vapor during the annealing process. This water splitting was theoretically predicted by Xu at al. [29] to have an impact on both the chemical and electronic structure of the examined films. (This is to be discussed below). Of course one can state that the irreversibility of contamination is only a matter of annealing temperature but we state that a further increase of the annealing temperature could lead to layer damage by uncontrolled oxygen desorption [30]. The crucial point is that, for all of the RGVO SnO_2_ nanolayers, we were considerably far from the ideal SnO_2_ stoichiometry.

**Figure 3:**
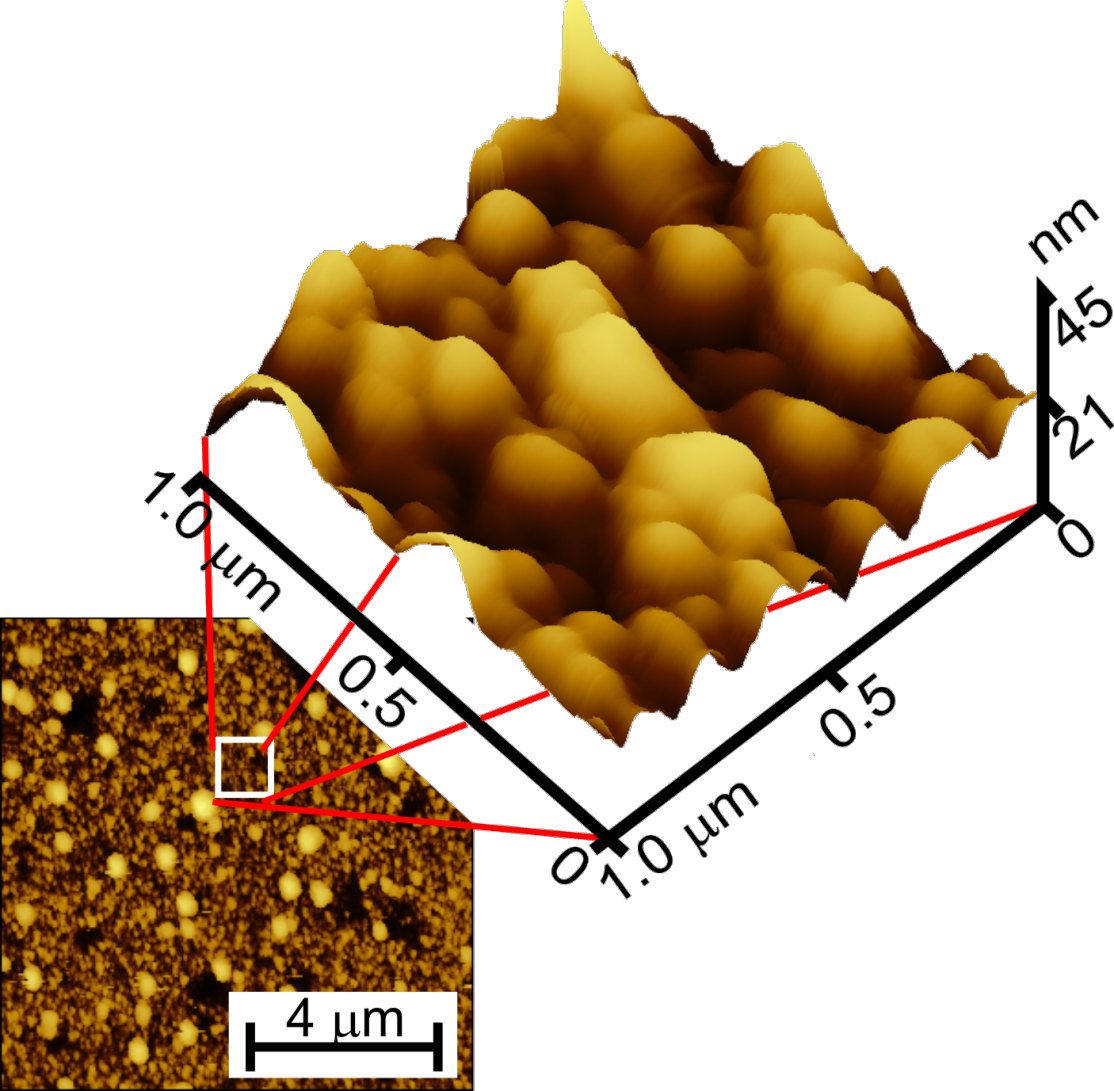
10 × 10 µm^2^ surface scan of SnO_2_ nanolayers presented together with 1 × 1 µm^2^ magnification. The white square presents the magnified area taken for 1 × 1 µm^2^ scan.

The diffusion of species from the ambient air is more than probable because the surface of the layers was highly developed and of high roughness. In Figure 3, an AFM 10 × 10 µm^2^ surface scan is presented together with a 1 × 1 µm^2^ magnification. The layer consisted of a number of more or less spherical nanograins of diameters within the range of 100–150 nm. The RMS roughness was equal to 6.1 nm while the surface area calculated over 1 × 1 µm^2^ scan was 1.017 µm^2^. The magnified image of the surface together with quantified surface data suggests that the surface layer of the SnO_2_ film is porous, which enables the diffusion of gas species from the ambient air.

**Figure 4:**
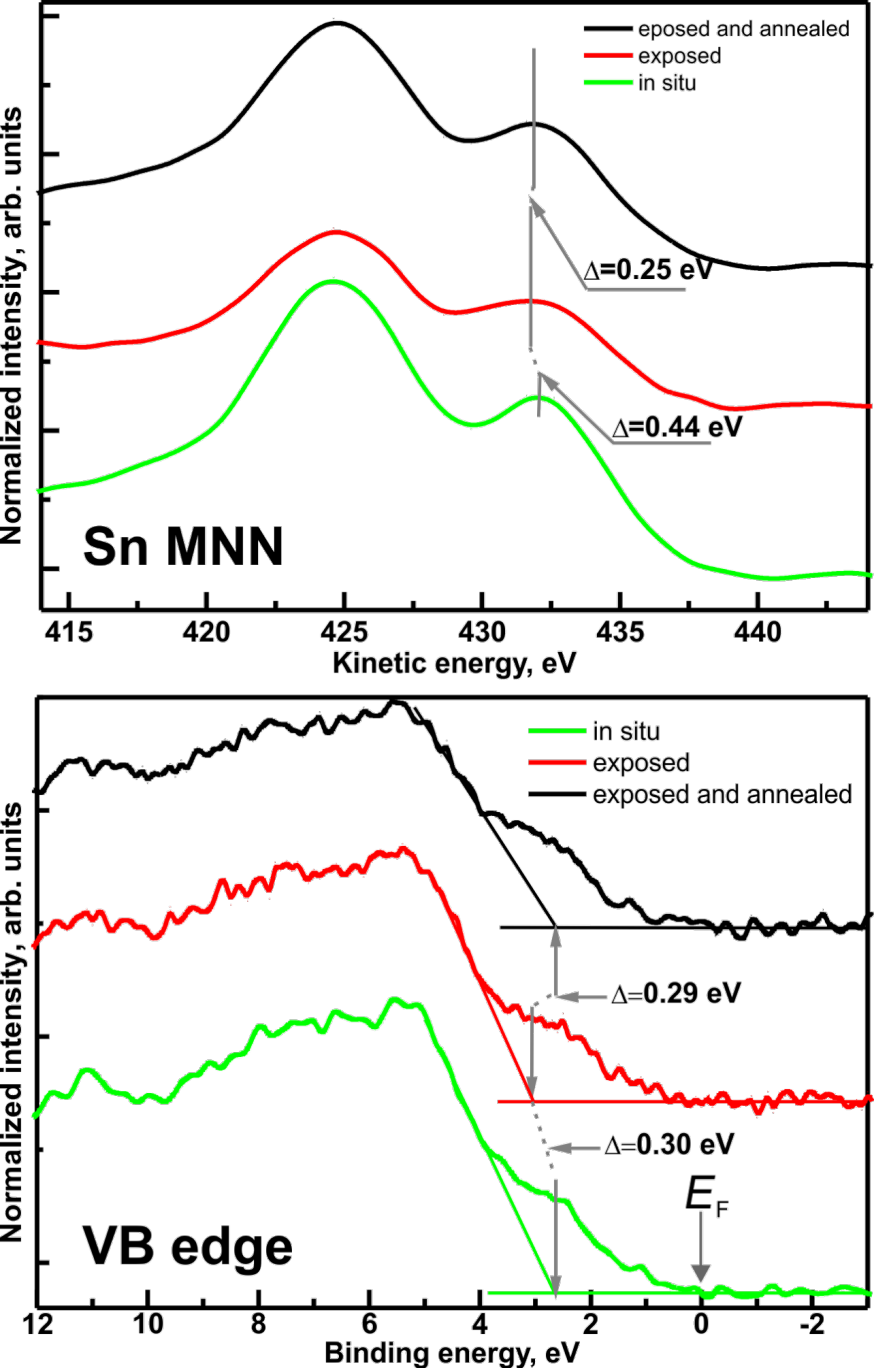
(a) Auger Sn MNN energy region recorded for pristine (in situ) and air-exposed SnO_2_ layers. The shift of the characteristic SnO_2_ transition is well visible (marked with grey vertical lines). (b) Valence band (VB) region of XPS spectra for pristine (“in situ”), air-exposed and annealed samples. The characteristic shape of the SnO_2_ valance region can be recognized [32].

SnO and SnO_2_ have similar binding energies in the Sn 3d region. However, an additional discrimination of the oxidation levels can be carried out using the XPS valence band spectrum and the Auger alpha parameter, which is based on the Auger MNN transition [31]. The shift of the Sn MNN transitions is shown in Figure 4a. The main kinetic energy peaks were from the M4N45N45 and M5N45N45 transitions and their fine structure was due to the different two-hole final states [30]. The separation of the two main peaks corresponded to the 3d spin–orbit splitting measured in XPS.

The chemical state of the examined layers determined from the energy difference between a representative XPS peak and a suitable Auger peak was defined as [33–34]


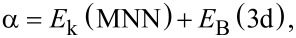


where *E*_K_(MNN) is the kinetic energy of the Auger transition MNN, and *E*_B_(3d) is the binding energy of an electron on the atomic level Sn 3d_5/2_. The maxima of the corresponding Auger lines (marked with short vertical lines in Figure 4a) were determined by fitting with Gaussian curves.

In general, a lower α indicates a lower electron density at the Sn atom, i.e., a higher oxidation state [31]. In our case the value of alpha changed from 919.14(15) eV in case of pristine samples to 918.73(15) eV for exposed samples. This meant a substantial increase of the oxidation state of the examined layers, which is consistent with the findings from XPS measurements.

A change in the oxidation state of tin also led to a change in the relation between tin and oxygen valence states originating from the mixing of the O 2p and Sn 5s orbitals [35–36]. The consequence is a shift of the valence band (VB) edge toward higher binding energies as shown in Figure 4b, which means an increase of the energy distance *E*_F_ − *E*_V_ assuming a common Fermi level of the analyzer and the sample setup.

The characteristic shape [32] of the spectrum line for SnO_2_ was shifted by ca. 0.30 eV after exposure to air, which confirmed a more n-type nature of the exposed SnO_2_. The effect could be attributed to the physical adsorption of atmospheric oxygen and/or water vapor at the surface of RGVO SnO_2_ nanolayers. Such adsorbates are often creating so-called surface dipoles [37] influencing the local charge distribution and, in consequence, the energy distance between the Fermi level position and the top of valence band at the surface, *E*_F_ − *E*_V_. This statement is justified because the observed in stoichiometry are not supposed to significantly influence the band gap value [38]. The statement is additionally supported by the results of air-exposed and UHV-annealed samples. After annealing the VB edge moved toward the initial energetic position (the shift was about 0.29 eV) so the difference to pristine samples was within the error of the measurement. Although the resolution of evaluating the valence band region is significantly lower than that of the most accurate photoemission yield spectroscopy [39–41], the magnitude of change is indisputable. This is of importance for the energy level alignment with regard to SnO_2_-based sensing devices. It also shows that for sensor devices based on changes of the surface conductivity (resistive sensors) the oxygen uptake from ambient air is affecting the energy band structure. However, the process is reversible by de-gassing, which proves the ability of SnO_2_ layers to restore under working conditions.

**Figure 5:**
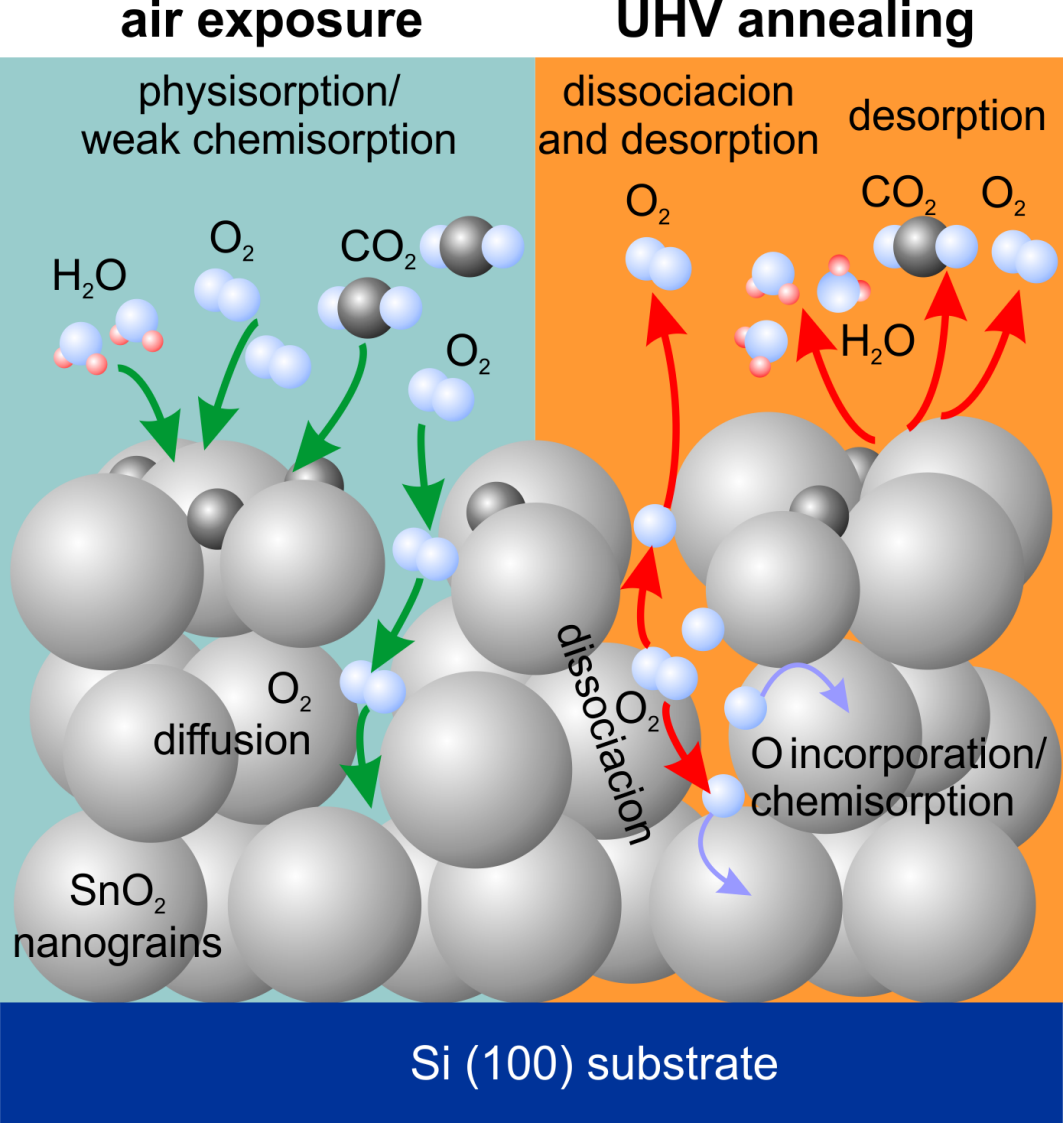
Scheme of the proposed process.

Taking into account the above statements as well as the discussion related to Figure 2 and the findings from the analysis of Figure 4a we propose that, most likely, during air exposure most of the oxygen is adsorbed on the sample surface while some amount of oxygen is diffusing towards deeper regions of the sample. During the exposure also water- and carbon-related contaminations are being adsorbed. During annealing, the diffused oxygen is partially desorbed together with surface oxygen and residual water/carbonaceous species, while part of it dissociates due to the elevated temperatures and is incorporated into the SnO_2_ layer. This permanently changes the stoichiometry of the layer. A schematic of the proposed process is depicted in Figure 5.

## Conclusion

In the study presented here, the impact of air exposure and subsequent UHV annealing on the surface chemistry of RGVO SnO_2_ nanolayers was examined. XPS results showed an increase of relative [O]/[Sn] concentration from 1.62 to 1.80 after air exposure. After UHV annealing the relative concentration was again reduced to 1.65 almost reaching the value of the pristine samples. The decomposition of the main core lines together with Auger alpha parameter analysis confirmed that the layers consisted of a mixture of SnO_2_ and SnO with significant domination of SnO_2_ after air exposure. The UHV annealing reinforced the SnO_2_ domination and suggested oxygen diffusion towards the deeper regions of the porous SnO_2_ nanolayers. The porosity of the nanolayers was confirmed by AFM investigation. The XPS data analysis performed for SnO_2_ nanolayers after UHV annealing suggested that most of the ambient–layer interactions during air exposure were based on physisorption or weak chemisorption. The air exposure process caused an increase of the water and carbon contaminations, which were in their majority desorbed during UHV annealing. Hence, our studies proved that the SnO_2_ nanolayers are not susceptible to significant air-induced contaminations.

Moreover, the electronic properties of RGVO nanolayers were changed upon air exposure as detected by analysis of the valence band edge XPS region. The relative position of Fermi level with respect to the top of the valence band at the surface (*E*_F_ − *E*_V_) increased by about 0.30 eV indicating a more n-type nature of the air-exposed RGVO nanolayers with respect to the pristine samples. This effect was found to be fully reversible during UHV annealing. The last effect is of significant importance for application of SnO_2_ nanolayers in resistive sensors and the prediction of their recovery behavior.

## References

[1] Carpenter M A, Mathur S, Kolmakov A (2012). Metal oxide nanomaterials for chemical sensors.

[2] Nelson J (2003). The Physics of Solar Cells (Properties of Semiconductor Materials).

[3] Ihokura K, Watson J (1994). The Stannic Oxide Gas Sensor: Principles and Applications.

[4] Sberveglieri G (1995). Sens Actuators, B.

[5] Göpel W, Schierbaum K D (1995). Sens Actuators, B.

[6] Barsan N, Schweitzer-Barberich M, Göpel W (1999). Fresenius' J Anal Chem.

[7] Manifacier J C, De Murcia M, Fillard J P, Vicario E (1977). Thin Solid Films.

[8] Park S-S, Zheng H, Mackenzie J D (1995). Mater Lett.

[9] Reddy M V, Tse L Y, Bruce W K Z, Chowdari B V R (2015). Mater Lett.

[10] Comini E, Faglia G, Sberveglieri G, Comini E, Faglia G, Sberveglieri G (2009). Electrical-Based Gas Sensing. Solid State Gas Sensing.

[11] Eranna G (2012). Metal oxide nanostructures as gas sensing devices.

[12] Kwoka M, Krzywiecki M (2015). Mater Lett.

[13] Sberveglieri G (1992). Sens Actuators, B.

[14] Sberveglieri G, Faglia G, Groppeli S, Nelli P, Camanzi A (1990). Semicond Sci Technol.

[15] Szuber J, Uljanow J, Karczewska-Buczek T, Jakubik W, Waczyński K, Kwoka M, Kończak S (2005). Thin Solid Films.

[16] Ottaviano L, Kwoka M, Bisti F, Parisse P, Grossi V, Santucci S, Szuber J (2009). Thin Solid Films.

[17] Dieguez A, Romano-Rodriguez A, Morante J R, Sangaletti L, Depero L E, Comini E, Faglia G, Sberveglieri G (2000). Sens Actuators, B.

[18] Lindau I, Pianetta P, Yu K Y, Spicer W E (1976). Phys Rev B.

[19] Kwoka M, Ottaviano L, Koscielniak P, Szuber J (2014). Nanoscale Res Lett.

[20] Wagner C D, Riggs W M, Davis L E (1979). Handbook of X-ray Photoelectron Spectroscopy.

[21] Watts J F, Wolstenholme J (2003). An Introduction to Surface Analysis by XPS and AES.

[22] (2017). Gwyddion – Documentation.

[23] Krzywiecki M, Grządziel L, Juszczyk J, Kaźmierczak-Bałata A, Erbe A, Bodzenta J (2014). J Phys D: Appl Phys.

[24] (2017). NIST X-ray Photoelectron Spectroscopy (XPS) Database Main Search Menu.

[25] Vrňata M, Myslík V, Vysloužil F, Jelínek M, Lančok J, Zemek J (2000). Sens Actuators, B.

[26] Marikutsa A V, Rumyantseva M N, Lada V Y, Gaskov A M (2010). J Solid State Chem.

[27] Kwoka M, Ottaviano L, Passacantando M, Santucci S, Czempik G, Szuber J (2005). Thin Solid Films.

[28] Choi D-w, Park J-S (2014). Surf Coat Technol.

[29] Xu H, Zhang R Q, Ng A M C, Djurišić A B, Chan H T, Chan W K, Tong S Y (2011). J Phys Chem C.

[30] Batzill M, Diebold U (2005). Prog Surf Sci.

[31] Futsuhara M, Yoshioka K, Takai O (1998). Thin Solid Films.

[32] (2017). Thermo Scientific XPS: Knowledge Base.

[33] Wagner C D (1978). J Vac Sci Technol (N Y, NY, U S).

[34] Krzywiecki M, Grządziel L, Sarfraz A, Iqbal D, Szwajca A, Erbe A (2015). Phys Chem Chem Phys.

[35] Walsh A, Payne D J, Egdell R G, Watson G W (2011). Chem Soc Rev.

[36] Krzywiecki M, Sarfraz A, Erbe A (2015). Appl Phys Lett.

[37] Grządziel L, Krzywiecki M, Peisert H, Chassé T, Szuber J (2011). Thin Solid Films.

[38] Sanon G, Rup R, Mansingh A (1991). Phys Rev B.

[39] Szuber J, Grządziel L (2000). Thin Solid Films.

[40] Kwoka M, Ottaviano L, Passacantando M, Czempik G, Santucci S, Szuber J (2006). Appl Surf Sci.

[41] Kwoka M, Ottaviano L, Szuber J (2012). Appl Surf Sci.

